# Exploring cognitive load in anatomy education: a study of 3D virtual reality and 2D learning environments using fNIRS

**DOI:** 10.3389/fpsyg.2026.1767614

**Published:** 2026-05-05

**Authors:** Erika Johannessen, Rebecca Benjamin, Paul Hungler, Prithila Angkan, Adam Szulewski

**Affiliations:** 1 Department of Chemical Engineering, Queen’s University, Kingston, ON, Canada; 2 School of Medicine, Queen’s University, Kingston, ON, Canada; 3 Department of Electrical and Computer Engineering, Queen’s University, Kingston, ON, Canada; 4 Department of Emergency Medicine and Psychology, Queen’s University, Kingston, ON, Canada

**Keywords:** anatomy education, cognitive load, fNIRS, machine learning, medical education, virtual reality

## Abstract

**Introduction:**

Although virtual reality (VR) is increasingly deployed as a tool for education, little is known about how learning modality influences cognitive processing or whether wearable neuroimaging technologies can accurately classify cognitive load in this context.

**Methods:**

In this study, 21 first-year medical students were randomly assigned to learn cardiac anatomy using either traditional 2D materials (*n* = 10) or immersive 3D VR (*n* = 11). Prefrontal hemodynamic activity was continuously monitored using functional near-infrared spectroscopy (fNIRS), while cognitive load was assessed across three learning periods using the Klepsch questionnaire. Learning outcomes were evaluated through pre- and post-learning assessments.

**Results:**

Results demonstrated that both groups had comparable knowledge gains and reported similar levels of intrinsic, extraneous, and germane cognitive load. In contrast, fNIRS revealed modality-related differences in neural dynamics: learners using 2D materials showed significantly longer time-to-peak oxygenation across multiple channels, with large effect sizes (Cohen’s d = 1.18–1.66). Deep learning classifiers successfully distinguished high from low cognitive load using fNIRS features under leave-one-subject-out validation, with extraneous load achieving the strongest performance (91% F1-score and 92% accuracy), followed by intrinsic cognitive load (63% F1-score and 73% accuracy) and total cognitive load (60% F1-score and 78% accuracy).

**Discussion:**

Together, these findings highlight the value of combining neurophysiological and subjective measures and demonstrate the feasibility of fNIRS-based cognitive load classification, offering a foundation for future adaptive and learner-responsive instructional systems in medical education.

## 1 Introduction

Anatomy is widely recognized as a foundational pillar of medical education, essential for building the clinical competence required for effective patient care (Turney, 2007). However, students frequently struggle to develop adequate spatial understanding of complicated three-dimensional anatomical structures from traditional two-dimensional learning materials such as textbooks, atlases, and PowerPoint presentations (Triepels et al., 2020). This challenge is particularly pronounced in cardiac anatomy, where complex spatial relationships between chambers, valves, and vessels must be accurately conceptualized for clinical applications. As medical curricula continue to face pressures from expanding content and reduced contact hours, there is a critical need for innovative instructional technologies that can optimize anatomical learning efficiency while ensuring deep, transferable knowledge acquisition (García-Robles et al., 2024).

Virtual reality (VR) has emerged as a promising technology for medical education, offering immersive three-dimensional visualizations that may facilitate spatial learning of anatomical structures (Moro et al., 2017; García-Robles et al., 2024). The appeal of VR extends beyond pedagogical benefits; traditional cadaver-based anatomy instruction faces significant logistical constraints including limited availability and high costs, as well as ethical concerns among students regarding the use of human remains for educational purposes (Nawras et al., 2023). Unlike traditional two-dimensional representations, VR environments allow learners to interact with anatomical models from multiple perspectives, potentially reducing the cognitive processing demands associated with mentally constructing spatial relationships from flat images. Meta-analyses have found that 3D visualization tools can enhance both factual and spatial anatomical knowledge compared to traditional methods (Yammine and Violato, 2015), although findings remain inconsistent across studies (Triepels et al., 2020). Students consistently report positive perceptions and high motivation when using VR for anatomy learning, suggesting these tools may offer benefits beyond measurable performance outcomes (Walsh et al., 2025). Nevertheless, questions persist regarding whether the advantages of VR in anatomy education stem from enhanced spatial encoding, reduced cognitive load, or simply increased engagement and novelty.

Cognitive load theory (CLT) provides a theoretical framework for understanding how instructional design affects learning by considering the inherent limitations of working memory (Sweller, 1988). The theory distinguishes three types of cognitive load: intrinsic load, arising from the inherent complexity of the material to be learned; extraneous load, imposed by suboptimal instructional design; and germane load, devoted to schema construction and learning processes (Sweller et al., 1998). In medical education, CLT has gained substantial recognition as a guiding principle for curriculum design, particularly given that many clinical activities require simultaneous integration of multiple knowledge domains that can exceed learners’ working memory capacity (Szulewski et al., 2021). When applied to anatomy instruction, CLT suggests that learning modalities requiring extensive mental transformation of 2D representations into 3D may impose additional extraneous load, potentially leaving fewer cognitive resources available for meaningful learning. Conversely, 3D and VR environments may reduce transformation demands by presenting anatomical structures in formats that more closely match their real-world configuration.

Functional near-infrared spectroscopy (fNIRS) has emerged as a valuable non-invasive neuroimaging modality for investigating cognitive processes in educational contexts (Pinti et al., 2020). By measuring changes in oxygenated and deoxygenated hemoglobin concentrations in cortical tissue, fNIRS provides an indirect measure of neural activity with several practical advantages over other neuroimaging techniques (Fishburn et al., 2014). For example, compared to functional magnetic resonance imaging (fMRI), fNIRS offers greater ecological validity due to its portability, tolerance to movement artifacts, and compatibility with naturalistic task environments, making it particularly valuable for studying learning in realistic educational settings like clinical simulation (Ayaz et al., 2012). The prefrontal cortex, accessible to fNIRS measurement, is centrally involved in working memory, executive function, and effortful cognitive processing, making it an appropriate tool for cognitive load assessment (Herff et al., 2014; Meidenbauer et al., 2021). Previous research has demonstrated that fNIRS measures of prefrontal activation correlate with task difficulty and self-reported cognitive load across various learning tasks (Causse et al., 2017; Fishburn et al., 2014).

Recent advances in machine learning have opened new possibilities for classifying cognitive states from neurophysiological signals (Khan et al., 2024). Both traditional machine learning algorithms and deep learning approaches have been applied to fNIRS data, with varying degrees of success depending on the classification task, feature extraction methods, and validation strategies employed (Gado et al., 2023; Karmakar et al., 2023; Yu et al., 2025). Convolutional neural networks (CNNs) and multilayer perceptrons (MLPs) have shown particular promise for extracting meaningful patterns from hemodynamic time series data (Ma et al., 2021). However, a critical consideration for practical applications is whether classification models can generalize to new individuals not encountered during training—a challenge that requires rigorous person-independent validation approaches such as leave-one-subject-out cross-validation (Kwon and Im, 2021). The ability to accurately classify cognitive load states from fNIRS signals could enable real-time adaptive learning systems that adjust instructional difficulty based on learner state, representing a significant advance for personalized education.

While research has separately examined VR-based anatomy education and fNIRS-based cognitive load assessment, no studies to date have integrated these approaches to investigate how learning modality influences both neural activation patterns and cognitive load classification performance. The present study addresses this gap by comparing prefrontal cortex hemodynamic responses in first-year medical students learning cardiac anatomy via traditional 2D materials versus immersive 3D VR. We collected concurrent fNIRS recordings and self-reported cognitive load measures using the validated Klepsch questionnaire across three learning periods. To evaluate whether fNIRS signals can reliably distinguish cognitive load states, we implemented both traditional machine learning algorithms and deep learning architectures, assessing generalization through person-independent cross-validation. This approach allowed us to examine three key questions: (1) whether 2D and 3D learning modalities produce distinct temporal patterns of prefrontal activation as measured by fNIRS, (2) how self-reported cognitive load compares between modalities, and (3) whether machine learning models can accurately classify cognitive load states from hemodynamic features in a manner that generalizes to new learners.

## 2 Methodology

The study was reviewed and approved by the Health Sciences and Affiliated Teaching Hospitals Research Ethics Board (HSREB) at Queen’s University (File No. 6042008). All participants provided written informed consent prior to participation.

### 2.1 Participants

Twenty-one first-year medical students from the Queen’s University School of Medicine participated in the study. At the time of participation, students had received no formal instruction in cardiac anatomy. This population was selected to ensure relative homogeneity in educational background and baseline anatomy experience. Although demographic characteristics such as age, gender distribution, and prior VR familiarity were recorded (Table 1), participants were broadly similar in academic stage and training level, reducing the likelihood that differences in prior educational experience influenced the observed results. A convenience sample was recruited via class announcement. Participants were randomly assigned to either the 3D learning group (*n* = 11) or the 2D learning group (*n* = 10) using simple random allocation without stratification. Randomization was intended to distribute individual differences, including prior anatomy experience and familiarity with VR, across groups. Baseline anatomy knowledge was assessed prior to the learning session, and no significant differences were observed between groups. Prior familiarity with VR was also recorded and was present in both groups (Table 1). Formal spatial ability was not directly measured, and therefore its potential influence cannot be fully excluded.

**Table 1:** Participant demographics by group.

Demographic	2D group (*N* = 11)	3D group (*N* = 10)
First language	English (*N* = 9), Other (*N* = 1)	English (*N* = 11)
Other language	Hebrew (*N* = 1)	N/A
Gender	Woman (*N* = 6), Man (*N* = 4)	Woman (*N* = 8), Man (*N* = 3)
Age	Mean = 23.4, SD = 1.9	Mean = 24.1, SD = 2.3
Diagnosed mental health conditions	No (*N* = 8), Yes (*N* = 2)	No (*N* = 10)
General level of anxiety	Medium (*N* = 5), High (*N* = 3), Low (*N* = 2)	Low (*N* = 5), Medium (*N* = 5), Very low (*N* = 1)
Attention deficits	No (*N* = 8), Yes (*N* = 2)	No (*N* = 10), Yes (*N* = 1)
Memory disorders	No (*N* = 10)	No (*N* = 10)
Medications for cognitive or attention disorders	No (*N* = 10)	No (*N* = 11)
Head injuries	No (*N* = 8), Yes (*N* = 2)	No (*N* = 9), Yes (*N* = 2)
Neurological conditions	No (*N* = 10)	No (*N* = 11)
Visual conditions	No (*N* = 10)	No (*N* = 10), Yes (*N* = 1)
Sleep quality	Well (*N* = 4), Poorly (*N* = 3), Very well (*N* = 2), Very poorly (*N* = 1)	Poorly (*N* = 4), Well (*N* = 4), Very well (*N* = 3)
Handedness	Right (*N* = 10)	Right (*N* = 11)
Substance use	Coffee/caffeine (*N* = 7), Coffee/caffeine, alcohol (*N* = 1)	Coffee/caffeine (*N* = 4), Alcohol (*N* = 1), Coffee/caffeine, alcohol (*N* = 1)
Familiarity with VR	No (*N* = 5), Yes (*N* = 5)	Yes (*N* = 8), No (*N* = 3)
VR comfort level	Neutral (*N* = 7), Comfortable (*N* = 3)	Comfortable (*N* = 4), Neutral (*N* = 4), Uncomfortable (*N* = 3)

### 2.2 Data collection

**Figure 1:**
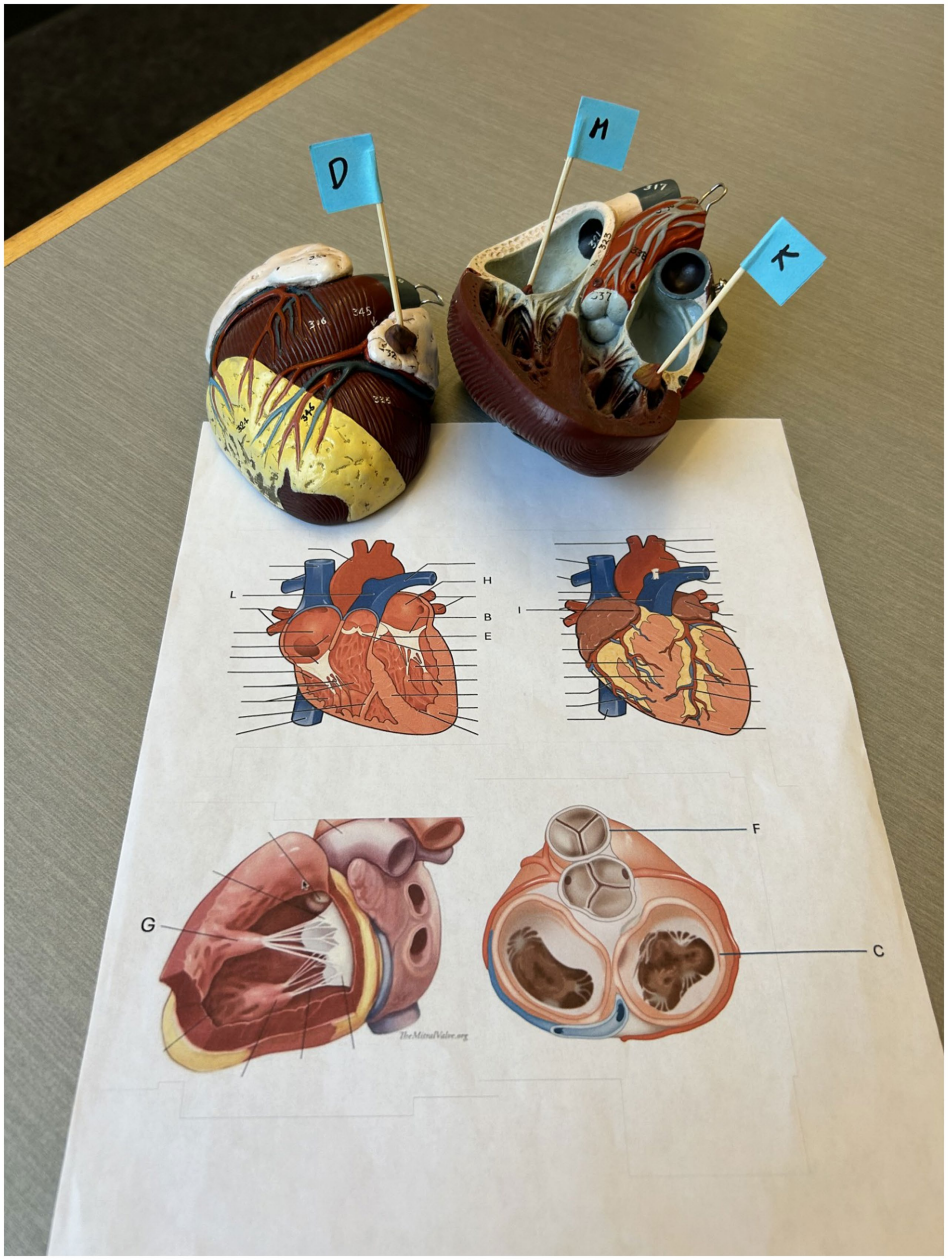
Cardiac anatomy knowledge assessment. A pre- and post-learning knowledge assessment was used to evaluate learning and included both 2D and 3D components.

All study sessions took place in the virtual reality room at the Clinical Simulation Centre at Queen’s University. Each participant first completed a pre-learning cardiac anatomy assessment to confirm no baseline differences between groups and to establish a reference point for evaluating learning gains. The assessment included both two-dimensional and three-dimensional components. Participants were presented with a series of cardiac images and plastic models with arrows indicating specific structures and were asked to identify them in writing (Figure 1). They were informed that their scores would be kept confidential and that no feedback would be provided in order to prevent learning effects before the study task.

**Figure 2:**
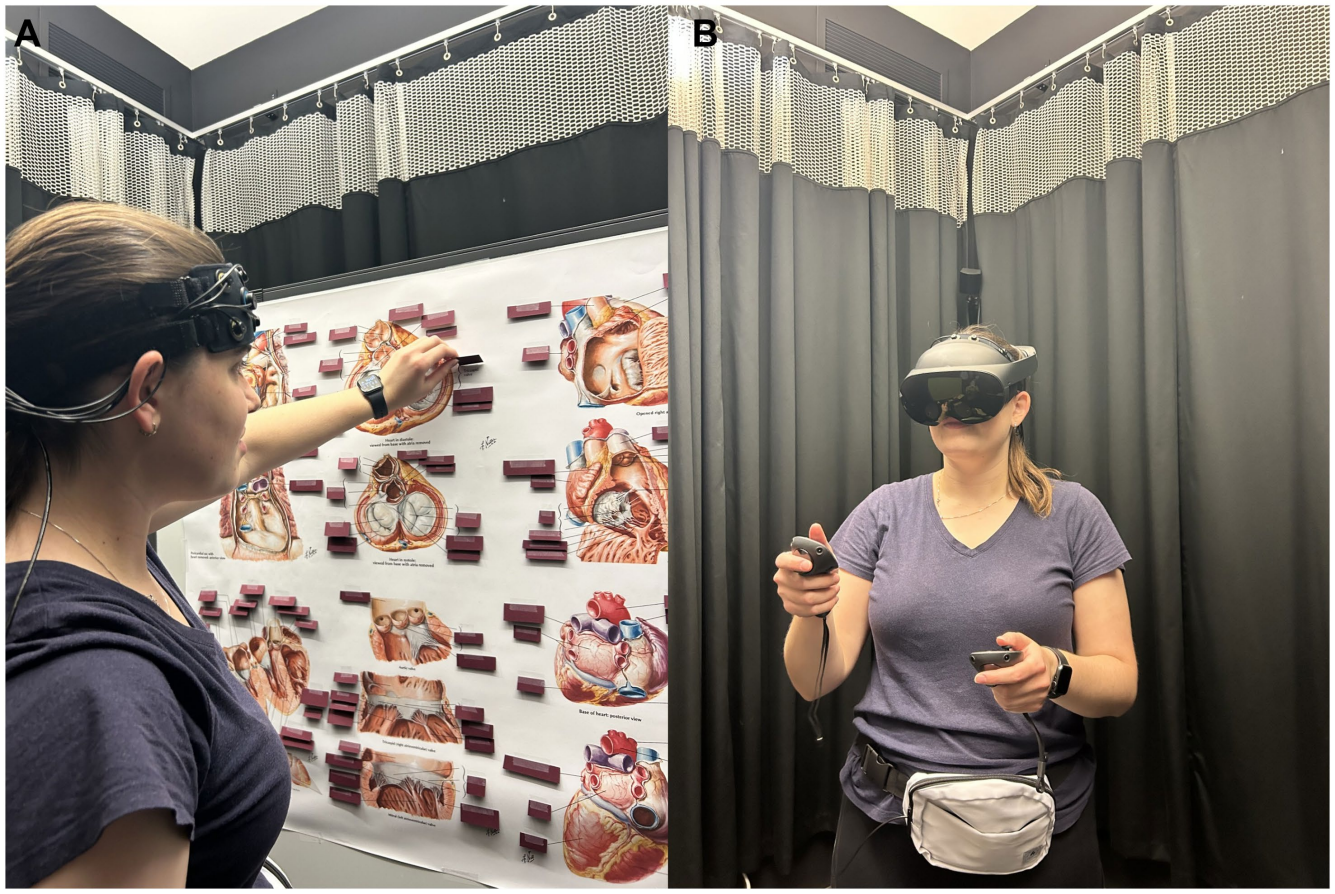
Cardiac anatomy learning modalities. **(A)** 2D poster, **(B)** 3D virtual reality.

After completing the pre-learning assessment, participants were familiarized with their assigned learning modality and were encouraged to ask any questions. Participants in the 3D learning group used the Human Anatomy VR software on a Meta Quest Pro headset and completed the built-in tutorial to learn the device’s controls. Participants in the 2D learning group studied a 48″ × 36″ color poster featuring cardiac diagrams adapted from Netter’s Atlas of Human Anatomy (8th Edition) (Figure 2). To approximate the upper limb interactivity of the VR environment, the anatomical labels on the poster were covered by paper flaps that had to be lifted to reveal the structure names.

Before beginning the learning session, the researchers introduced the Klepsch Cognitive Load Questionnaire, a validated instrument that differentiates between the three subtypes of cognitive load (Klepsch et al., 2017). Participants rate items on a 7-point Likert scale, with higher scores indicating greater cognitive load. Participants were asked to read the questionnaire and invited to request clarification if needed. The questionnaire was administered verbally periodically throughout the task rather than in written form, as removing the VR headset to complete a paper scale would have disrupted fNIRS optode-scalp contact and compromised data quality by introducing signal artifacts. Following orientation to the Klepsch questionnaire, the researchers then positioned the fNIRS headband on the participant’s forehead. The eight-channel system (OctaMon, Artinis Medical, Netherlands) continuously monitored changes in oxygenated (O₂Hb) and deoxygenated (HHb) hemoglobin within the prefrontal cortex using a fixed forehead-mounted optode configuration. This placement reflects both technical and physiological considerations. Technically, fNIRS measurement is limited to superficial cortical regions, and the forehead provides optimal optical access due to minimal hair interference and more consistent optode–scalp contact, improving signal reliability. Physiologically, the prefrontal cortex plays a central role in working memory, attentional control, and cognitive load regulation during complex learning tasks, making it an appropriate target region for investigating modality-related differences in cognitive processing. For participants in the 3D group, the VR headset was fitted over the fNIRS device once the sensors were in place.

Each participant completed a two-minute baseline recording while seated and fixating on a blank wall. The learning phase then began and consisted of three 6-min intervals, for a total of 18 min of self-directed study for both groups. Participants were instructed to explore the assigned learning material on cardiac anatomy at their own pace while standing. fNIRS signals were recorded throughout the session, and the learning task was paused every 6 min so that a researcher could verbally administer the Klepsch questionnaire. Participants’ responses were reported verbally. At the end of the learning period, the researcher assisted participants in removing the fNIRS headband and, when applicable, the VR headset. Participants then completed a post-learning anatomy assessment. Two test versions (A and B) were used, and participants were randomly assigned to receive one version before learning and the other afterward to control for order effects.

### 2.3 fNIRS preprocessing

Hemodynamic time series for oxygenated (O₂Hb) and deoxygenated (HHb) hemoglobin were preprocessed using a Python pipeline incorporating the MNE-Python, NumPy, and SciPy libraries (Gramfort et al., 2014). Data were structured within an MNE RawArray object containing 16 channels (eight O₂Hb / HHb pairs) sampled at 25 Hz. Motion artifacts were identified using an adaptive thresholding algorithm that computed the local mean and standard deviation within a ± 750-sample sliding window (30 s), with points exceeding three standard deviations flagged as artifacts. Flagged segments were corrected using cubic spline interpolation (SciPy UnivariateSpline, *k* = 3), with splines fit only to non-artifact data and evaluated at artifact time points to generate physiologically plausible reconstructions; the resulting artifact mask was applied consistently across all channels (Scholkmann et al., 2010). Subsequently, all channels were low-pass filtered using a second-order Butterworth filter with a 0.1 Hz cutoff frequency to suppress high-frequency noise arising from cardiac pulsations, respiration, and other physiological sources, thereby isolating slower hemodynamic fluctuations associated with task-related neural activity (Ghouse et al., 2023). Baseline correction was performed by computing the mean low-pass filtered signal during the two-minute resting baseline period for each channel and subtracting this value from the full time series to obtain baseline-normalized traces representing task-evoked hemodynamic changes relative to each participant’s physiological baseline. Finally, five statistical features were extracted from each channel: average (Avg), peak amplitude (Peak), time-to-peak (TimeToPeak), area under the curve (AUC), and variability, resulting in a total of 80 features per sample. These signal features were calculated using a sliding window approach with a window length of 20 s and an overlap of 50%, resulting in one set of features for each 10 s of data. The 20-s window length was selected to capture the full rise and partial recovery of the hemodynamic response, and the 50% overlap was chosen to increase sample density while limiting redundancy between adjacent windows, consistent with common practice in fNIRS feature extraction (Aghajani et al., 2017). After preprocessing and removing samples with missing values, the final dataset consisted of 2,356 samples across all participants.

### 2.4 Analysis

The primary objective of the machine learning analysis was to predict cognitive load states from fNIRS-derived hemodynamic features. Two categories of classification approaches were evaluated: traditional machine learning algorithms and deep learning neural networks. All models used the 80 fNIRS features (statistical measures of oxygenated and deoxygenated hemoglobin signals) as input to predict binary cognitive load states (high vs. low). This dual approach enabled comparison between interpretable, feature-based methods and more complex neural architectures capable of learning hierarchical representations from fNIRS hemodynamic patterns.

#### 2.4.1 Cross-validation strategy

Both leave-one-subject-out (LOSO) and 5-fold person-independent cross-validation strategies were employed to evaluate model generalization to unseen individuals. For LOSO cross-validation, the model was tested on one participant while training on all remaining participants; this process was repeated for each participant, and average accuracy and F1-scores were calculated across all iterations. For 5-fold cross-validation, the 21 participants were randomly shuffled using a fixed random seed (42) to ensure reproducibility, then divided into five groups. Each fold used one group as the test set, comprising 4–5 participants (approximately 450–550 samples), while the training set contained 16–17 participants (approximately 1,800–1,900 samples). This approach ensured that no participant appeared in both training and test sets within any fold, providing a realistic assessment of model performance on new individuals. Person-independent evaluation is essential for real-world deployment of cognitive load monitoring systems, as it simulates scenarios where the system must generalize to users not encountered during training. It should be noted that the 2,356 samples used for model training and evaluation are not independent observations, as they were derived from continuous fNIRS time series using overlapping sliding windows (20s windows, 50% overlap) across 21 participants. Consequently, adjacent samples share data and exhibit temporal autocorrelation within each participant.

#### 2.4.2 Traditional machine learning algorithms

Six classical machine learning algorithms were evaluated as baseline classifiers. The Support Vector Machine (SVM) was configured with a radial basis function (RBF) kernel, regularization parameter C = 1.0, and automatic gamma scaling. Extreme Gradient Boosting (XGBoost) employed an ensemble of 100 decision trees with a maximum depth of 5, providing robust performance through iterative gradient-based optimization and regularization to prevent overfitting. The Random Forest (RF) classifier similarly used an ensemble of 100 decision trees trained independently using bootstrap aggregation and random feature selection at each split, reducing variance through averaging of multiple uncorrelated models. Logistic Regression (LR) served as a linear baseline classifier with a maximum of 1,000 iterations for convergence. The K-Nearest Neighbors (KNN) classifier used *k* = 5 neighbors for prediction, making decisions based on local similarity patterns in the feature space without explicit model training. Finally, Gaussian Naive Bayes (GNB) implemented a probabilistic approach assuming feature independence and Gaussian distributions within each class.

Hyperparameters for all models were specified *a priori* based on established defaults and values commonly reported in the fNIRS and broader neuroimaging classification literature, rather than through data-driven optimization. Specifically, the SVM regularization parameter (C = 1.0) with an RBF kernel reflects standard default settings; ensemble sizes of 100 trees for both XGBoost and Random Forest are widely adopted in neuroimaging studies; and the deep learning configurations (learning rate = 0.001, dropout *p* = 0.3, batch size = 32) represent best-practice starting values for small neuroimaging datasets. All hyperparameters were fixed prior to cross-validation, and no tuning was performed on held-out participants, thereby avoiding data leakage and ensuring unbiased estimates of model generalization.

#### 2.4.3 Deep learning neural networks

To capture more complex relationships in fNIRS hemodynamic responses, two deep learning architectures were implemented: a multilayer perceptron (MLP) and a convolutional neural network (CNN), both designed for binary classification of cognitive load from the 80-dimensional fNIRS feature vector.

The MLP architecture consisted of an input layer with 80 neurons corresponding to the fNIRS features, followed by two hidden layers with 128 and 64 neurons, respectively. The output layer consisted of a single neuron with sigmoid activation for binary classification probability. Each hidden layer incorporated batch normalization, followed by Rectified Linear Unit (ReLU) activation to introduce non-linearity, and dropout (*p* = 0.3) to prevent overfitting. The network was trained using binary cross-entropy loss and optimized using the Adam optimizer with a learning rate of 0.001.

The CNN architecture was designed to learn local patterns and feature combinations from the 80-dimensional feature vector by treating it as a one-dimensional sequence. Input features were reshaped from a flat vector of 80 values to a tensor of shape (batch_size, 1, 80). The first convolutional layer used 32 filters with kernel size 3, followed by batch normalization, ReLU activation, max-pooling with stride 2, and dropout (*p* = 0.3). The second convolutional layer used 64 filters with identical batch normalization, ReLU activation, max-pooling, and dropout operations. Following the convolutional blocks, feature maps were flattened and passed through two fully connected layers of 128 and 1 neurons, respectively, with batch normalization, ReLU activation, and dropout before the final sigmoid activation for binary classification. Both networks were trained for 100 epochs with a batch size of 32 samples, implemented using PyTorch, and trained on an NVIDIA GeForce RTX 2080 Ti GPU.

#### 2.4.4 Cognitive load labels

Model performance was evaluated across four cognitive load metrics derived from participant self-reports. Intrinsic cognitive load and extraneous cognitive load were measured on 1–7 scales and binarized using the same thresholds of 5 for both measures. For both extraneous and intrinsic cognitive load, average values of 1–5 were classified as low cognitive load (Class 0) and average values of 6–7 as high cognitive load (Class 1). This threshold was selected to distinguish clearly elevated cognitive load from lower and moderate levels while maintaining sufficient class representation for machine learning analysis. Total cognitive load, representing the summation of intrinsic and extraneous cognitive load across all measurement items, was measured on a scale of 0–56 and binarized at a threshold of 22 to maintain adequate class representation. These thresholds were fixed *a priori* and were not optimized through data-driven exploration; as such, alternative criterion may yield different class distributions and influence classification performance, a consideration for future work.

#### 2.4.5 Evaluation metrics

Model performance was evaluated using both accuracy and macro-averaged F1-score. The macro-averaged F1-score computes the harmonic mean of precision and recall for each class separately, then averages these scores, treating both classes equally regardless of their frequency in the dataset. This metric is particularly valuable for imbalanced datasets as it prevents models from achieving artificially high performance by simply predicting the majority class. For each model and fold, both metrics were computed on the test set, and mean and standard deviation values were calculated across all five folds.

## 3 Results

### 3.1 Participant characteristics

Twenty-one first-year medical students participated in this study (*N* = 10 in 2D group, *N* = 11 in 3D group). Participants had a mean age of 23.4 years (SD = 1.9) in the 2D group and 24.1 years (SD = 2.3) in the 3D group, with the majority being women (60% in 2D, 73% in 3D), right-handed, and native English speakers. Complete demographic and baseline characteristics are presented in Table 1.

### 3.2 Self-reported cognitive load

**Figure 3:**
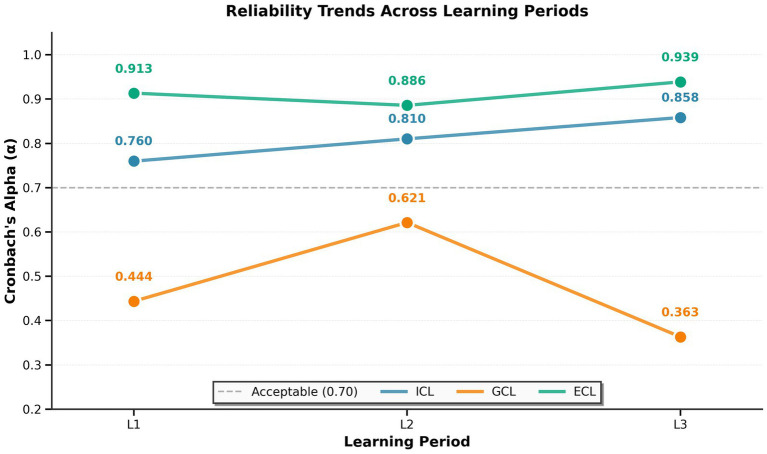
Reliability of cognitive load subscales. Cronbach’s alpha for intrinsic (ICL, blue), germane (GCL, orange), and extraneous (ECL, green) cognitive load subscales across three learning periods. Dashed line shows acceptable reliability threshold (α = 0.70). ICL and ECL demonstrated acceptable to excellent reliability, while GCL consistently fell below acceptable levels, indicating poor measurement quality.

Cronbach’s alpha coefficients were calculated for each cognitive load subscale at each learning period to assess internal reliability of the Klepsch psychometric scale (Tavakol and Dennick, 2011). The intrinsic cognitive load (ICL) subscale demonstrated good reliability, with Cronbach’s α ranging from 0.760 (acceptable) in Period 1 to 0.858 (good) in Period 3, showing improvement across learning periods (mean α = 0.809 ± 0.049). The extraneous cognitive load (ECL) subscale exhibited excellent and highly consistent reliability across all three periods, with α values ranging from 0.923 to 0.928 (mean α = 0.926 ± 0.003). All ECL items showed high inter-item correlations (*r* > 0.80), indicating exceptional measurement quality. In contrast, the germane cognitive load (GCL) subscale showed problematic reliability across all learning periods, with Cronbach’s α ranging from 0.363 (unacceptable) in Period 3 to 0.621 (questionable) in Period 2 (mean α = 0.476 ± 0.132) (Figure 3). These low alpha values suggest that the GCL items may not be measuring a single, unified construct. As a result, subsequent analyses therefore focused on the ICL and ECL subscales of the Klepsch questionnaire, which demonstrated better psychometric properties and clear construct coherence.

**Figure 4:**
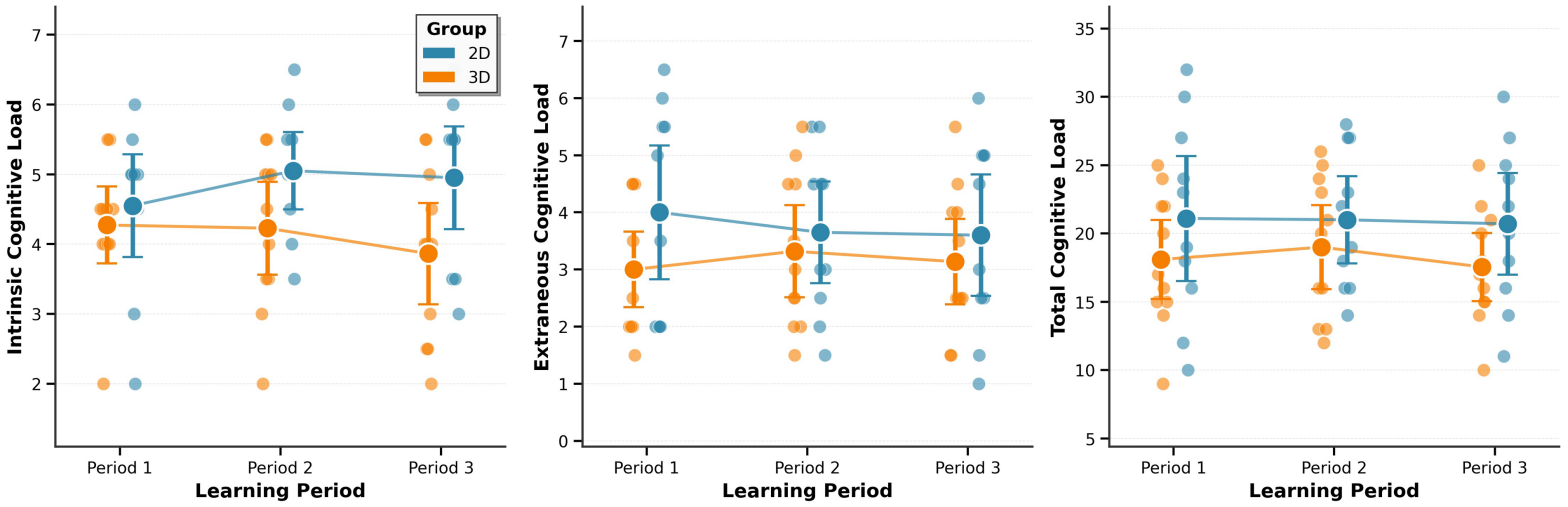
Self-reported cognitive load across three learning periods. Self-reported cognitive load remained stable across all periods for both groups (repeated measures ANOVAs: all *p* > 0.05).

Self-reported cognitive load demonstrated stability across all three learning periods for both groups. Descriptive statistics for intrinsic, germane, extraneous, and total cognitive load revealed minimal variation throughout the learning session, with overlapping confidence intervals across time points within each group (Figure 4).

For intrinsic cognitive load, the 2D group reported mean scores of 4.55 (95% CI: 3.81–5.29) in Period 1, 5.05 (95% CI: 4.50–5.61) in Period 2, and 4.95 (95% CI: 4.21–5.69) in Period 3. The 3D group showed similar stability with means of 4.27 (95% CI: 3.72–4.82) in Period 1, 4.23 (95% CI: 3.56–4.89) in Period 2, and 3.86 (95% CI: 3.14–4.59) in Period 3. Extraneous cognitive load was consistently lower than intrinsic load, with the 2D group averaging 4.00 (95% CI: 2.83–5.17), 3.65 (95% CI: 2.76–4.54), and 3.60 (95% CI: 2.54–4.66), and the 3D group averaging 3.00 (95% CI: 2.34–3.66), 3.32 (95% CI: 2.51–4.13), and 3.14 (95% CI: 2.39–3.89) across the three periods. Total cognitive load showed minimal variation, with 2D group means of 21.10 (95% CI: 16.52–25.68), 21.00 (95% CI: 17.81–24.19), and 20.70 (95% CI: 16.97–24.43), and 3D group means of 18.09 (95% CI: 15.19–20.99), 19.00 (95% CI, 15.93–22.07), and 17.55 (95% CI, 15.05–20.04) across the three periods. Complete cognitive load ratings by participant and learning period are available in Supplementary Table S2.

Repeated measures ANOVAs were conducted to test for changes across all three learning periods. For the 2D group, no significant changes were observed in intrinsic load (*F*(2, 18) = 3.073, *p* = 0.071, η^2^G = 0.041), extraneous load (*F*(2, 18) = 1.751, *p* = 0.202, η^2^G = 0.012), or total cognitive load (*F*(2, 18) = 0.119, *p* = 0.889, η^2^G = 0.001). Similarly, the 3D group showed no significant changes in intrinsic load (*F*(2, 20) = 1.496, *p* = 0.248, η^2^G = 0.029), extraneous load (*F*(2, 20) = 0.894, *p* = 0.425, η^2^G = 0.012), or total cognitive load (*F*(2, 20) = 1.227, *p* = 0.314, η^2^G = 0.017). Effect sizes were negligible to small across all comparisons (all η^2^G < 0.05), further supporting the interpretation that self-reported cognitive load remained essentially unchanged throughout the learning experience.

Between-group comparisons showed no clear differentiation in self-reported cognitive load. While the 2D group showed numerically higher mean scores on intrinsic and extraneous load dimensions compared to the 3D group, these differences were small and fell within overlapping confidence intervals.

### 3.3 Learning performance outcomes

#### 3.3.1 Total assessment score

**Figure 5:**
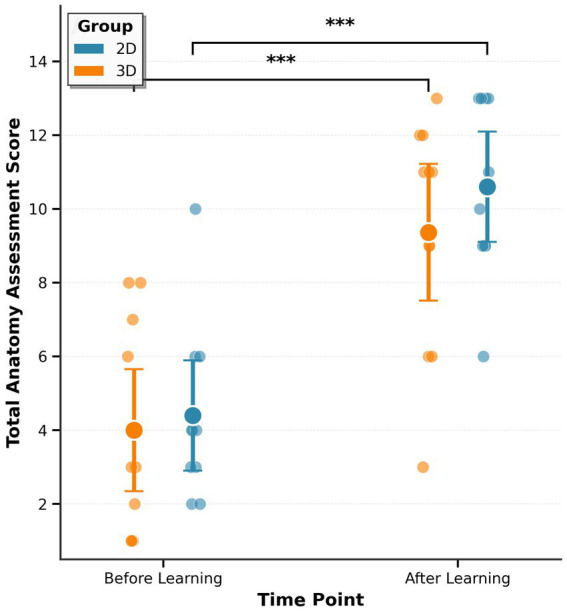
Anatomy assessment performance before and after learning. Total assessment scores for 2D (blue, *n* = 10) and 3D (orange, *n* = 11) groups before and after learning. Individual points show participants; large circles show means ± SEM. Both groups showed significant improvement (***p < 0.001). Upper bracket indicates between-group comparison at post-learning.

Prior to the learning session, both groups demonstrated similar baseline anatomy knowledge (Figure 5). The 2D group achieved a mean total score of 4.40 (95% CI: 2.90–5.90) out of 20 possible points, while the 3D group scored 4.00 (95% CI: 2.35–5.65), with no significant difference between groups (*t*(19) = 0.301, *p* = 0.767). Individual participant scores are provided in Supplementary Table S1. Baseline performance was similarly low across question modalities, with both groups scoring between 26 and 38% correct on 2D questions and 30–38% correct on 3D questions. No significant differences were observed between groups for either 2D questions (*t*(19) = −0.135, *p* = 0.894) or 3D questions (*t*(19) = 0.763, *p* = 0.455), confirming successful random assignment and equivalent starting knowledge. The low baseline scores in both groups suggest that most learners were encountering this material for the first time, minimizing the likelihood that prior anatomy expertise influenced modality-related differences in neurophysiological or behavioral outcomes.

Following the learning session, both groups demonstrated substantial improvement in anatomy knowledge. The 2D group achieved a mean total score of 10.60 (95% CI: 9.10–12.10), representing an improvement of 6.20 points (95% CI: 4.90–7.50) from baseline. The 3D group scored 9.36 (95% CI: 7.51–11.22), an improvement of 5.36 points (95% CI: 3.55–7.18). Despite the numerically higher performance in the 2D group, this difference did not reach statistical significance (*t*(19) = 1.004, *p* = 0.328), indicating comparable learning effectiveness between the two modalities. However, post-learning scores approached the upper range of the assessment scale, raising the possibility that ceiling effects may have limited the ability to detect subtle differences between groups.

#### 3.3.2 Scores by question modality

**Figure 6:**
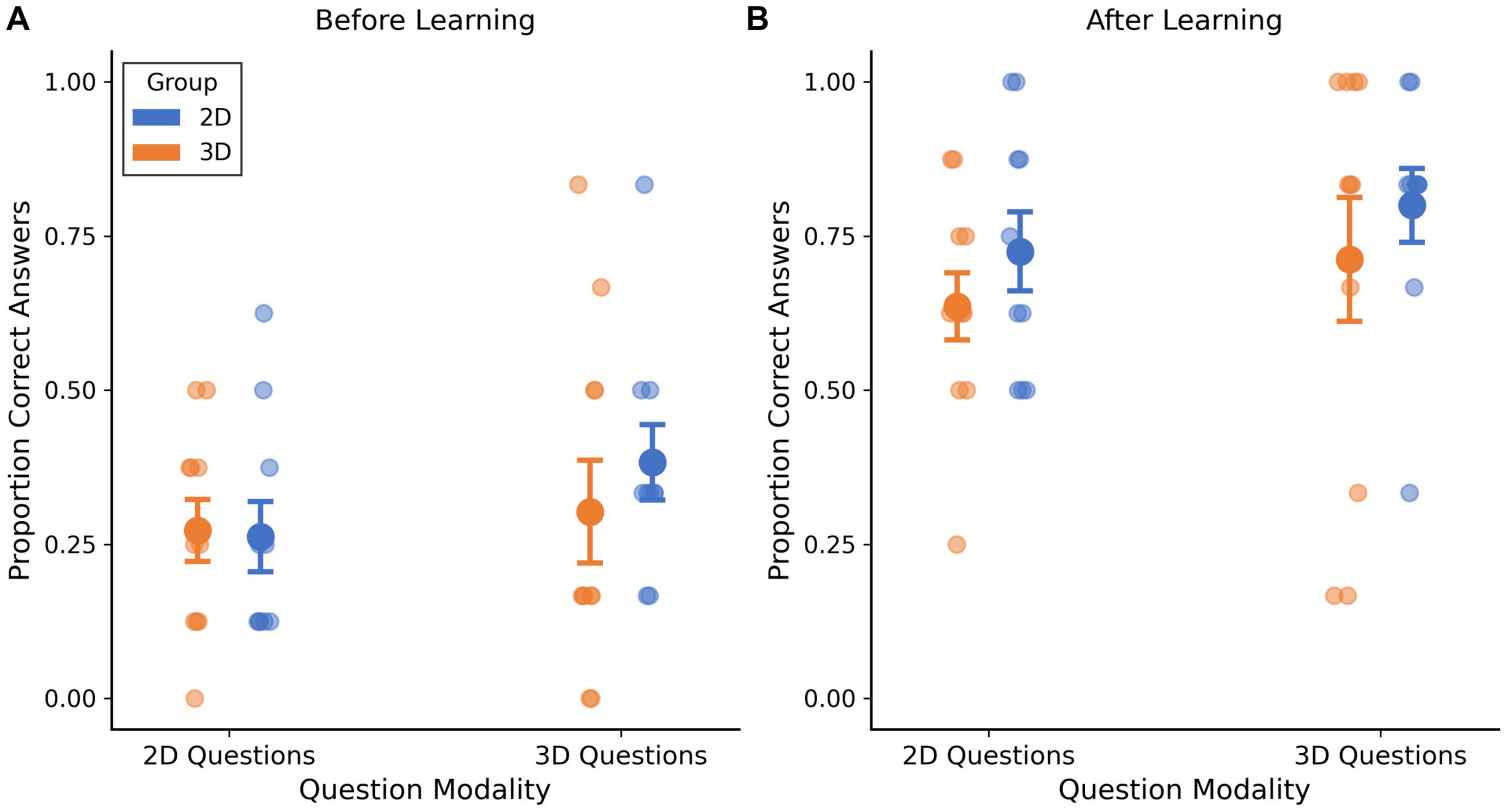
Performance by question modality proportion correct on 2D and 3D questions before **(A)** and after **(B)** learning. Points show individual participants; large circles show means ± SEM.

Both groups showed substantial gains on both 2D and 3D questions following learning (Figure 6). For 2D questions, the 2D group improved from 26.3% correct (95% CI: 15.0–37.5%) at baseline to 72.5% correct (95% CI: 60.0–85.0%) post-learning, an increase of 46.3 percentage points (95% CI: 35.3–57.2). The 3D group improved from 27.3% correct (95% CI: 17.5–37.1%) to 63.6% correct (95% CI: 53.0–74.3%), an increase of 36.4 percentage points (95% CI: 26.2–46.5). The difference between groups in post-learning performance on 2D questions was not statistically significant (*t*(19) = 1.060, *p* = 0.302). For 3D questions, the 2D group improved from 38.3% correct (95% CI: 26.4–50.3%) at baseline to 80.0% correct (95% CI: 68.3–91.7%) post-learning, an increase of 41.7 percentage points (95% CI: 28.6–54.8). The 3D group improved from 30.3% correct (95% CI: 13.9–46.7%) to 71.2% correct (95% CI: 51.5–91.0%), an increase of 40.9 percentage points (95% CI: 19.6–62.2). Again, no significant difference was observed between groups in post-learning performance on 3D questions (*t*(19) = 0.731, *p* = 0.474).

Notably, both groups performed well on both question types following learning, with no evidence of modality-specific advantages. The 2D learning group achieved comparable performance on 3D questions (80.0% correct) as on 2D questions (72.5% correct), while the 3D learning group similarly showed no disadvantage on 2D questions (63.6% correct) compared to 3D questions (71.2% correct). This pattern suggests that both learning modalities effectively supported knowledge transfer across question formats.

### 3.4 fNIRS signal metrics: group comparisons

**Figure 7:**
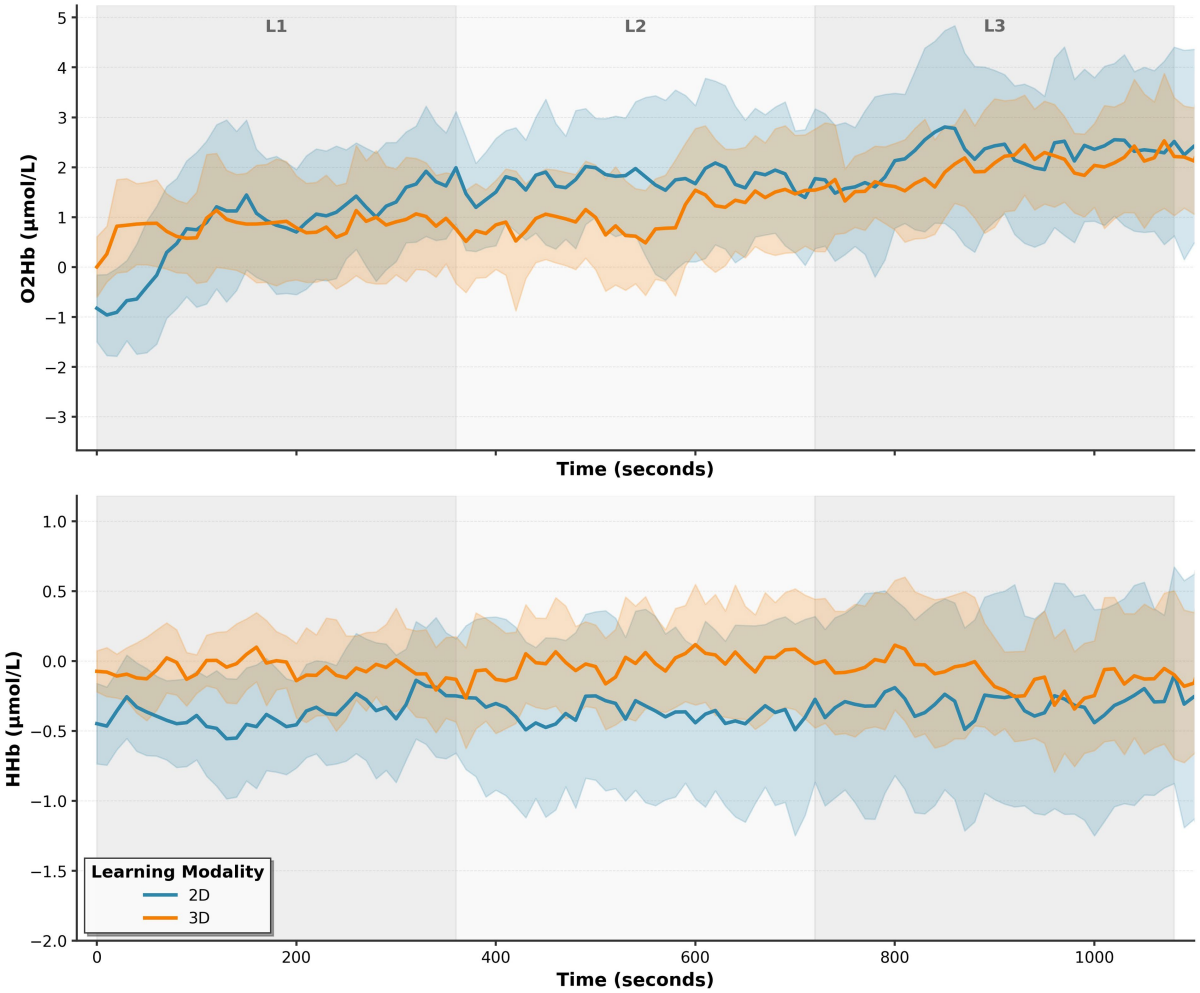
Prefrontal hemodynamic responses across learning periods. Group-averaged oxygenated (O_2_Hb, top) and deoxygenated (HHb, bottom) hemoglobin concentrations across the three 6-min learning periods (L1, L2, L3) for 2D (blue) and 3D (orange) groups. Solid lines represent group means; shaded regions indicate 95% confidence intervals. Both groups showed increasing O_2_Hb over the learning session, with the 2D group exhibiting numerically higher values and greater inter-subject variability.

Group-averaged hemodynamic responses across the full learning session are shown in Figure 7. Both groups demonstrated increasing oxygenated hemoglobin over time, with the 2D group exhibiting numerically higher values and greater inter-subject variability. To compare hemodynamic responses between learning modalities at the participant level, 80 signal metrics (baseline-corrected O₂Hb and HHb measurements including average concentration, peak amplitude, time to peak, area under the curve, and variability) were averaged for each participant across all learning periods. Independent samples *t*-tests or Mann–Whitney U tests were conducted based on normality assessments (Shapiro–Wilk test, α = 0.05), with effect sizes calculated using Cohen’s d.

**Figure 8:**
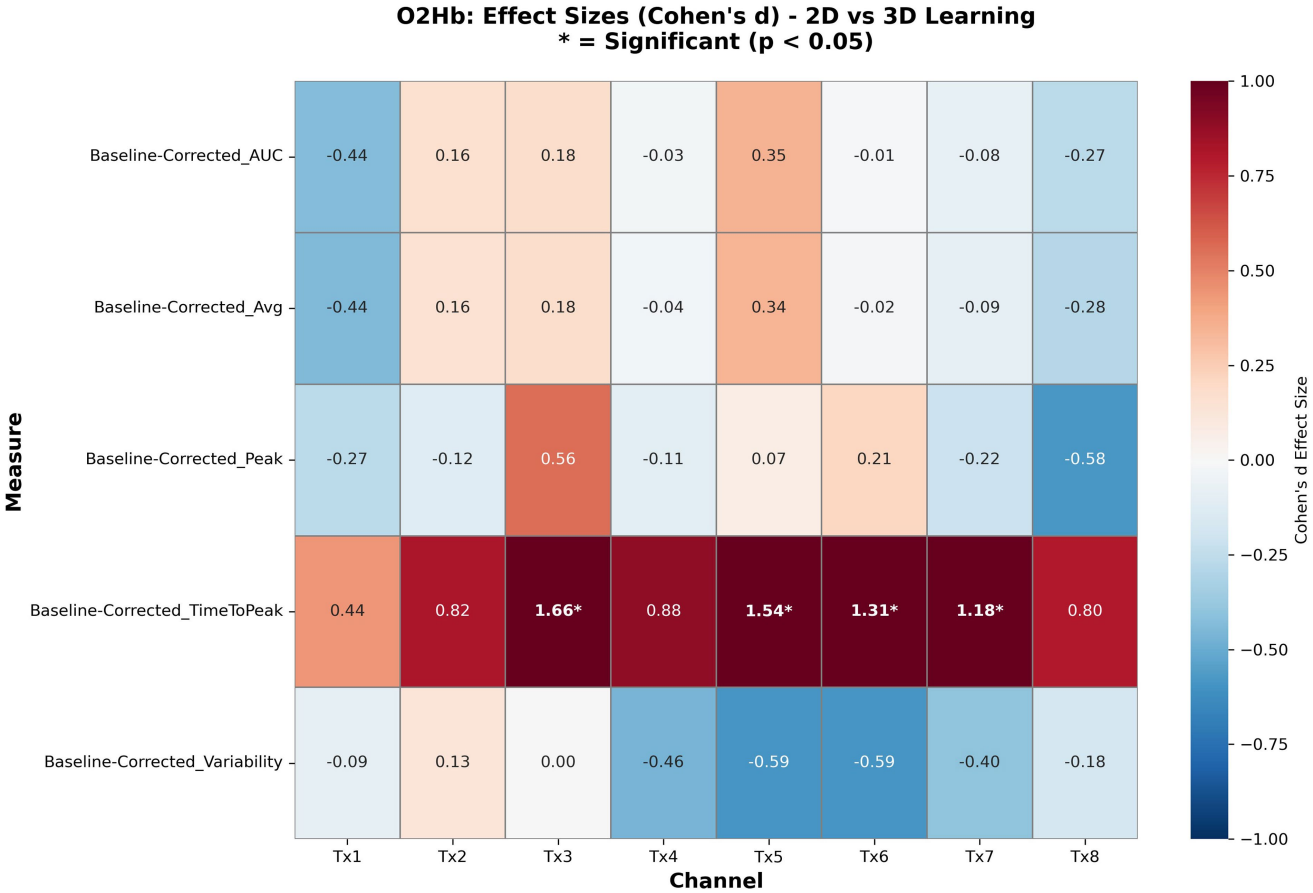
Heatmap of effect sizes (Cohen’s *d*) for oxygenated hemoglobin measures across all transmitter locations. Red indicates 2D > 03D; blue indicates 3D > 2D. Asterisks denote statistical significance (*p* < 0.05). The consistent pattern of positive effect sizes for time to peak (row 4) across transmitters 3, 5–7 demonstrates the robustness of delayed activation in 2D learning.

Of the 80 signal metrics examined, four demonstrated statistically significant differences between groups (*p* < 0.05), all relating to oxygenated hemoglobin time to peak activation (Figure 8). Complete participant-level signal statistics are provided in Supplementary Table S3. Notably, all four significant findings showed the same directional pattern, with the 2D learning group exhibiting longer latencies to peak oxygenation compared to the 3D/VR group, suggesting slower cognitive processing or less immediate engagement with the 2D materials. No correction for multiple comparisons was applied across the 80 signal metrics. However, the concentration of all four significant results within a single metric type and the consistently large effect sizes suggest these findings are unlikely to reflect chance inflation alone.

Additionally, all four comparisons demonstrated very large effect sizes (Cohen’s *d* > 1.0), indicating substantial practical significance beyond statistical significance. It should be noted, however, that effect size estimates based on small samples (*n* = 21) are subject to inflation, and these values should be interpreted with appropriate caution pending replication in larger cohorts. Nonetheless, mean differences were consistent, and the standard deviations (Table 2) indicate that time-to-peak responses varied across participants, suggesting that the observed delay in 2D learners reflects a general trend rather than a uniform effect across all individuals. No other signal metrics, including HHb measurements or alternative O2Hb parameters (average, peak amplitude, AUC, variability), reached statistical significance at the *p* < 0.05 threshold (Table 2).

**Table 2:** Group differences in time-to-peak O₂Hb responses for channels demonstrating significant effects.

Channel/electrode	2D Mean (SD) Time to peak (sec)	3D Mean (SD) Time to peak (sec)	Difference	*p*-value	Cohen’s d
Channel 3	5.27 (0.25)	4.88 (0.23)	+0.39 s	0.005	1.66**
Channel 5	5.32 (0.27)	4.90 (0.27)	+0.42 s	0.002	1.54**
Channel 6	5.34 (0.26)	4.96 (0.31)	+0.37 s	0.008	1.31**
Channel 7	5.14 (0.35)	4.78 (0.27)	+0.37 s	0.014	1.18**

Mean time-to-peak values (±SD) are reported for the 2D and 3D learning groups, along with absolute differences, *p*-values, and corresponding effect.

** denotes that the effect size is significant at the *p* < 0.01 level.

### 3.5 Machine learning findings

A key objective of this study was to determine whether fNIRS-derived hemodynamic responses could reliably classify cognitive load states during anatomy learning. We operationalized this as a binary classification task using fNIRS features as input to predict high versus low cognitive load states for three cognitive load dimensions: intrinsic cognitive load, extraneous cognitive load, and total cognitive load.

#### 3.5.1 5-fold cross-validation results

Average extraneous cognitive load was most accurately predicted from fNIRS features. MLP achieved the highest F1-score of 0.639 ± 0.222 with an accuracy of 0.900 ± 0.093. However, the discrepancy between accuracy and F1-score suggests class imbalance issues, with the majority class dominating predictions in some models. Traditional classifiers showed similar patterns, with accuracies reaching 0.740–0.911 but F1-scores ranging from 0.425 to 0.639.

Average intrinsic cognitive load was the second most accurately classified dimension. The MLP reached the highest F1-score (0.600 ± 0.190; accuracy = 0.724 ± 0.122) and the CNN performed slightly lower (F1-score = 0.573 ± 0.127; accuracy = 0.714 ± 0.087). Traditional machine learning classifiers performed substantially worse, with Logistic Regression achieving the strongest performance among them (F1-score = 0.502 ± 0.161; accuracy = 0.661 ± 0.146).

Total cognitive load was the most difficult dimension to classify from fNIRS signals. The deep learning models outperformed traditional algorithms, with MLP reaching an F1-score of 0.565 ± 0.113 and accuracy of 0.649 ± 0.133, followed by CNN with an F1-score of 0.544 ± 0.083 and accuracy of 0.637 ± 0.077. Among traditional machine learning algorithms, Logistic Regression performed best with an F1-score of 0.541 ± 0.165 and accuracy of 0.636 ± 0.158, followed by KNN (F1 = 0.529 ± 0.075, accuracy = 0.615 ± 0.127).

The top machine learning models for cognitive load classification with 5-fold cross-validation are shown in Table 3.

**Table 3:** Top machine learning models for cognitive load classification (5-fold cross-validation).

Classification target	Best model	F1-score	Accuracy
Average extraneous cognitive load	MLP	0.639 ± 0.222	0.900 ± 0.093
Average intrinsic cognitive load	MLP	0.600 ± 0.190	0.724 ± 0.122
Total cognitive load	MLP	0.565 ± 0.113	0.649 ± 0.133

#### 3.5.2 Leave-one-subject-out validation results

LOSO validation, which provides a more stringent test of whether fNIRS patterns can generalize to new individuals, yielded performance patterns consistent with 5-fold cross-validation, with extraneous load achieving the strongest classification and total load remaining the most challenging.

Average extraneous cognitive load achieved the strongest classification performance under LOSO validation. MLP achieved an F1-score of 0.914 ± 0.245 with accuracy of 0.916 ± 0.243, demonstrating excellent generalization to unseen subjects. Notably, the near-identical F1-score and accuracy indicate that the deep learning model effectively handled class distribution, avoiding the imbalance issues observed in the 5-fold validation. CNN also performed well with an F1-score of 0.833 ± 0.290 and accuracy of 0.909 ± 0.249. These results suggest that extraneous load produces reliable and distinctive neurophysiological patterns in the prefrontal cortex that deep learning models can successfully detect across individuals.

Average intrinsic cognitive load showed the second strongest classification performance. CNN reached an F1-score of 0.626 ± 0.303 and accuracy of 0.730 ± 0.259, while MLP achieved an F1-score of 0.620 ± 0.281 and accuracy of 0.745 ± 0.229. This ranking is consistent with the 5-fold results, suggesting that intrinsic load produces moderately distinct neurophysiological patterns that can generalize across participants.

Total cognitive load remained the most difficult dimension to classify under LOSO validation. Deep learning models achieved moderate performance, with MLP reaching an F1-score of 0.604 ± 0.296 and accuracy of 0.779 ± 0.255, followed by CNN with an F1-score of 0.579 ± 0.290 and accuracy of 0.762 ± 0.263. Traditional machine learning algorithms performed substantially worse, with Random Forest achieving the best F1-score among them (0.448 ± 0.305; accuracy = 0.645 ± 0.380). The top machine learning models for cognitive load classification with leave-one-subject-out validation are shown in Table 4. Complete results for all classifiers are available in Supplementary Table S4.

**Table 4:** Top machine learning models for cognitive load classification (leave-one-subject-out validation).

Classification target	Best model	F1-score	Accuracy
Average extraneous cognitive load	MLP	0.914 ± 0.245	0.916 ± 0.243
Average intrinsic cognitive load	CNN	0.626 ± 0.303	0.730 ± 0.259
Total cognitive load	MLP	0.604 ± 0.296	0.779 ± 0.255

#### 3.5.3 Cross-validation strategy comparison

The comparison between 5-fold and LOSO validation reveals that fNIRS-based cognitive load classification can generalize effectively to new individuals. These findings should be interpreted as exploratory with respect to real-world application, with deployment-related implications intended to motivate future work rather than indicate immediate practical readiness. Notably, LOSO validation achieved similar or higher mean F1-scores compared to 5-fold cross-validation across all cognitive load dimensions, with extraneous load showing the most dramatic improvement (0.639 to 0.914). However, LOSO validation resulted in considerably higher variability (standard deviations of 0.24–0.30 compared to 0.09–0.22 in 5-fold validation), reflecting substantial inter-individual differences in fNIRS response patterns. Deep learning neural networks (CNN, MLP) consistently outperformed traditional machine learning algorithms in LOSO validation, suggesting that neural networks better capture subject-invariant features in hemodynamic responses for predicting cognitive load across individuals. The maintained or improved mean performance under participant-independent validation, despite increased variability, suggests that fNIRS-based cognitive monitoring systems can generalize to new users without prior calibration, though individual differences in hemodynamic responses contribute to prediction uncertainty.

## 4 Discussion

### 4.1 Temporal dynamics of neural activation

A central finding of this study was the consistent delay in prefrontal hemodynamic response among 2D learners compared to 3D/VR learners. Within each standardized 10-s analysis windows, 2D learners required approximately 0.4 s longer to reach peak oxygenation across four prefrontal channels, with large effect sizes (*d* = 1.18–1.66) underscoring the robustness of this pattern. Given that the hemodynamic response typically unfolds over 5–7 s, this delay represents a meaningful proportion of the neural response window. The observed group difference in time-to-peak is interpretable within the technical constraints of fNIRS. Although the low-pass filter applied during preprocessing smooths the hemodynamic signal and limits the precision with which peak timing can be pinpointed, this effect applies equally across both groups and all channels. Because fNIRS captures slow neurovascular responses rather than rapid neural events, timing differences of this magnitude are best understood as reflecting relative differences in how quickly each group engaged prefrontal regions, rather than exact temporal measurements. The consistency of this pattern across four independent channels and the very large effect sizes (Cohen’s *d* = 1.18–1.66) support the conclusion that this difference reflects a genuine physiological signal.

We interpret this finding through the lens of cognitive load theory as a theoretical explanation. Learners studying 2D materials may require greater mental transformation to construct three-dimensional spatial representations from flat images, which cognitive load theory suggests could increase extraneous cognitive demands. Neuroimaging studies have shown that spatial transformation tasks such as mental rotation reliably engage prefrontal cortical regions, reflecting the executive and working memory processes required to manipulate spatial representations (Mutlu et al., 2020). However, it is important to emphasize that extraneous load was not experimentally manipulated in this study, and our conclusions are based on indirect indicators including fNIRS-derived hemodynamic responses, performance outcomes, and self-reported cognitive load rather than direct manipulation of cognitive load components. Reduced reliance on working memory for spatial manipulation is one plausible mechanism, but this remains an open hypothesis. In contrast, VR presents anatomical structures in formats congruent to their real-world configuration, reducing transformation demands and enabling more direct encoding pathways. The faster hemodynamic response in VR learners may therefore reflect differences in neural processing efficiency during spatial learning tasks. Within cognitive load theory, such differences are often associated with reduced working memory demands and more efficient schema acquisition. However, schema construction itself was not directly measured in this study and remains a theoretical interpretation rather than an empirically observed variable. These findings carry practical implications for medical education. Empirically, this study demonstrated modality-related differences in prefrontal hemodynamic timing despite equivalent learning outcomes and self-reported cognitive load. These results indicate that learning modality can influence neural processing dynamics even when behavioral performance and subjective cognitive load measures do not differ. However, the functional significance of these neurophysiological differences remains unclear.

### 4.2 Self-reported cognitive load

Despite observable differences in hemodynamic responses between learning modalities, self-reported cognitive load did not differ statistically between groups across any learning period. This divergence suggests that neurophysiological and subjective measures may be capturing different aspects of cognitive processing. fNIRS reflects underlying patterns of cortical activation associated with cognitive effort, whereas self-report instruments assess learners’ conscious perception and appraisal of that effort. Because subjective ratings rely on introspection and metacognitive awareness, they may not fully capture subtle or transient processing differences detectable at the neural level.

Rather than indicating superiority of one method over the other, this dissociation underscores the value of multimodal assessment approaches. Neurophysiological and self-report measures provide complementary perspectives on cognitive load, each offering insight into distinct but related dimensions of cognitive effort. Neither approach alone provides a complete representation of cognitive processing. Instead, integrating subjective reports with objective neural indicators enables a more comprehensive understanding of both experienced and underlying cognitive demands.

Importantly, fNIRS should not be viewed as a replacement for validated self-report instruments such as the Klepsch questionnaire. Self-report measures provide essential information about learners’ subjective experience, which cannot be directly inferred from neurophysiological data. Together, these approaches offer a more nuanced and balanced characterization of cognitive load in educational contexts.

### 4.3 Learning outcomes

Both learning modalities produced significant and comparable improvements in anatomy knowledge, with no statistically significant differences in post-learning assessment scores. Notably, knowledge transfer was not modality-locked: both groups performed well on both 2D and 3D test questions, suggesting that the underlying spatial understanding generalized across representational formats. Several factors may explain why distinct neural signatures did not translate into differential learning outcomes, including equivalent engagement across modalities, potential ceiling effects due to the limited scope of the anatomical content, and the short exposure duration preventing modality-specific advantages from fully emerging. Nevertheless, the combination of equivalent outcomes with faster prefrontal hemodynamic responses in VR raises important considerations. If VR achieves comparable learning through faster prefrontal recruitment, it might hypothetically preserve cognitive reserve for managing additional complexity or concurrent demands, a point of particular relevance in clinical training settings that require simultaneous integration of anatomical, diagnostic, and procedural skills. Whether such preserved capacity would benefit learners operating near their cognitive load threshold remains an open empirical question, and longitudinal studies with more demanding task conditions would be needed to test this hypothesis.

### 4.4 Machine learning for cognitive state classification

The machine learning analyses demonstrated that fNIRS-derived features can distinguish between high and low cognitive load with meaningful accuracy. Deep learning models achieved the strongest performance for extraneous cognitive load, reaching 91% F1-score and 92% accuracy under leave-one-subject-out validation, results that suggest real potential for adaptive learning systems capable of monitoring learner state in real time. Intrinsic and total cognitive load were classified with moderate accuracy (63 and 60% F1-scores, respectively), indicating that these dimensions produce less distinctive hemodynamic signatures.

The classification performance observed across cognitive load dimensions is theoretically consistent with both cognitive load theory and the neurophysiological properties of fNIRS. Extraneous cognitive load, which arises from instructional design, is closely linked to effortful executive control and working memory processes mediated by the prefrontal cortex, the primary region measured in this study. As a result, extraneous load likely produces more consistent prefrontal hemodynamic patterns across learners, supporting stronger classification. Intrinsic cognitive load, by contrast, reflects the inherent complexity of the learning material and learners’ prior knowledge, making it more internally driven and more variable across individuals. Although intrinsic load engages working memory resources, its neural expression may be distributed across additional cortical and subcortical regions involved in domain-specific processing, reducing the specificity of prefrontal fNIRS signals and limiting classification accuracy. For total cognitive load, which is an aggregate of the two, combining these dimensions likely reduces signal specificity and obscures neural patterns that are separable at the component level, thereby limiting classification performance. Importantly, cognitive load subtypes were not experimentally manipulated in this study. Instead, load classifications were derived from self-report measures, and fNIRS signals were used to classify these reported states. As such, neural classification findings reflect associations with reported load states rather than causal neural signatures of experimentally controlled load components.

Notably, LOSO validation achieved similar or higher mean F1-scores compared to 5-fold cross-validation across all cognitive load dimensions, with extraneous load showing the most substantial improvement (0.639–0.914). This suggests that fNIRS-based classification can generalize effectively to new individuals without prior calibration. However, LOSO validation resulted in considerably higher variability (standard deviations of 0.24–0.30 compared to 0.09–0.22 in 5-fold validation), reflecting substantial inter-individual differences in hemodynamic response patterns that contribute to prediction uncertainty.

The consistent superiority of CNN and MLP architectures over traditional machine learning algorithms suggests that hemodynamic data contain complex, nonlinear patterns that simpler models fail to capture. These findings point toward future applications in workload monitoring during simulation training and potential integration into medical education technologies, though further work with larger samples is needed to reduce individual variability and optimize model robustness.

### 4.5 Methodological considerations

This study offers several methodological contributions to the literature on neuroimaging in educational contexts. Data collection occurred under ecologically valid conditions (participants learned while standing and actively interacting with materials) rather than the constrained laboratory environments typical of neuroimaging research. The concurrent acquisition of fNIRS signals with a validated cognitive load instrument enabled direct comparison of neural and subjective measures within the same learning episodes. Additionally, the rigorous person-independent cross-validation strategy provides robust performance estimates relevant to real-world deployment scenarios.

Our psychometric analysis revealed consistently poor internal reliability for the germane cognitive load subscale across all three learning periods (α = 0.36–0.62), indicating that germane load was not measured consistently in this study. As a result, germane load was excluded from subsequent machine learning analyses and interpretation. This finding aligns with prior work suggesting that germane load may be difficult to measure reliably using self-report instruments and may not represent a distinct construct separable from intrinsic load. Because germane load could not be reliably quantified, our conclusions focus on intrinsic and extraneous cognitive load, which demonstrated acceptable reliability (α = 0.76–0.93) and clearer associations with neurophysiological measures.

### 4.6 Implications for anatomy education

These findings demonstrate that learning modality can influence neurophysiological activation patterns even when learning outcomes and subjective cognitive load are equivalent. This suggests that neurophysiological measures such as fNIRS may capture aspects of cognitive processing not reflected in behavioral or self-report measures alone. Both VR and traditional learning modalities supported effective anatomy learning in this study. The choice of modality should therefore be considered within the context of institutional resources, including equipment availability, infrastructure, cost, and faculty training requirements.

The observed differences in prefrontal hemodynamic timing further indicate that learning modality influences neural processing dynamics in ways not reflected in behavioral performance or self-report measures. One possible interpretation is that modality-related differences in spatial representation or attentional demands contribute to these neural patterns. However, cognitive load components were not experimentally manipulated in this study, and the specific mechanisms underlying these differences cannot be determined from the present data. As such, the functional significance of these neurophysiological differences remains unclear.

### 4.7 Limitations

Several limitations warrant consideration. First, although our sample of 21 participants aligns with practical constraints typical of neuroeducation research involving specialized equipment and a unique learner population, this modest size limits statistical power for detecting subtler effects and reduces the generalizability of findings to the broader medical student population. Second, ceiling effects on the knowledge assessment suggest that the learning task may not have been sufficiently challenging, and because task difficulty was not systematically varied, the study did not sample the full range of the cognitive load scale. This likely obscured performance differences between modalities that might emerge under more demanding conditions. Third, while the 18-min learning session produced measurable knowledge gains, it does not address long-term retention. Whether the neural efficiency advantages observed in VR learners translate into more durable learning over extended time periods remains unknown. Fourth, fNIRS measurement was restricted to the prefrontal cortex, precluding examination of other cortical regions involved in spatial processing, such as the parietal cortex. Although the prefrontal cortex is centrally involved in working memory and executive function, making it an appropriate region for assessing cognitive load, a fuller understanding of modality-related neural differences would require imaging beyond this area. fNIRS is confined to monitoring superficial cortical regions due to the limited penetration depth of near-infrared light, making it unsuitable for investigating subcortical structures such as the hippocampus, which is critical for spatial learning (Burgess et al., 2002; Yang and Wang, 2025). Assessing hippocampal involvement, as well as interactions across multiple cortical regions, would therefore require complementary neuroimaging modalities such as fMRI.

With 21 participants and 2,356 samples, the dataset is modest in size, particularly at the participant level, which limits the complexity of models that can be trained without overfitting. To mitigate this risk, regularization strategies including dropout (*p* = 0.3) and batch normalization were applied throughout the deep learning architectures. The relatively high standard deviations observed under leave-one-subject-out validation (0.24–0.30) likely reflect performance variability across participants, consistent with the limited sample size. Furthermore, the sliding window approach used to generate samples introduces temporal autocorrelation within participant data, as adjacent windows share overlapping signal segments. While person-independent validation prevents participant-level leakage, future work could explore non-overlapping windows or autocorrelation-aware modeling approaches to further address this.

Both self-report and neurophysiological measures also have inherent limitations. Self-report instruments rely on introspection and may be influenced by response bias, interpretation of questionnaire items, and variability in metacognitive awareness. Conversely, fNIRS provides indirect measures of neural activity and is subject to physiological noise, inter-individual variability, and limited spatial resolution. Moreover, neurophysiological signals do not directly capture the subjective experience of cognitive effort. As a result, neither modality alone provides a complete representation of cognitive load, and integrating subjective and neurophysiological measures offers a more comprehensive characterization of cognitive processing. The analysis of 80 signal metrics without correction for multiple comparisons (e.g., Bonferroni or FDR) increases the theoretical risk of Type I error. Future studies with larger samples should consider applying formal corrections to confirm the reliability of the time-to-peak findings reported here. Similarly, the thresholds used to binarize cognitive load scores were determined *a priori* based on practical considerations of class balance rather than empirical optimization, and alternative thresholds may influence classification outcomes.

Finally, cognitive load components were not experimentally manipulated in this study. Instead, cognitive load classifications were based on participant self-report measures and interpreted alongside neurophysiological and performance data. As a result, conclusions regarding intrinsic, extraneous, and germane load reflect theoretical interpretation within the framework of cognitive load theory rather than direct causal inference. Future studies that systematically manipulate load components will be necessary to establish causal relationships between instructional design, cognitive load types, and neurophysiological responses.

### 4.8 Future directions

Future research should extend these findings in several directions. First, longitudinal designs assessing retention at delayed intervals would clarify whether the faster prefrontal hemodynamic responses observed in VR learners translates into more durable knowledge over time, and whether they reflect neural efficiency, attentional differences, or other mechanisms. Second, systematically manipulating different types of cognitive load, such as varying intrinsic load through content complexity or extraneous load through instructional design or distraction, would help isolate the measurement differences. Third, integrating fNIRS with complementary measures such as eye-tracking or electrodermal activity would provide a richer characterization of learner state. Larger samples would also support more advanced deep learning architectures and more thorough hyperparameter optimization, potentially improving classification accuracy and generalizability. Finally, the most promising direction may be real-time adaptive instruction, in which fNIRS-detected cognitive load triggers adjustments to instructional difficulty or pacing. Such closed-loop systems could personalize learning in ways that static curricula cannot, though their feasibility and efficacy remain to be demonstrated.

## 5 Conclusion

This study provides neurophysiological evidence that 2D and 3D/VR anatomy learning, while producing equivalent knowledge gains and self-reported cognitive load, engage distinct temporal patterns of prefrontal activation. The consistently delayed hemodynamic response observed in 2D learners is consistent with the hypothesis that traditional materials impose additional cognitive transformation demands that remain below conscious awareness yet are detectable through fNIRS. These findings support the integration of neuroimaging methods alongside subjective measures for a more complete assessment of cognitive load in educational contexts. The successful classification of cognitive load states using deep learning models further demonstrates the feasibility of fNIRS-based monitoring systems that could eventually enable real-time adaptive instruction in medical education. As curricula continue to expand and instructional time remains limited, understanding the hidden neural costs of different learning modalities may inform more strategic decisions about when and how to deploy emerging educational technologies.

## Data Availability

The original contributions presented in the study are included in the article/Supplementary material, further inquiries can be directed to the corresponding author.

## Supplementary material

Supplementary Table S1 Pre- and post-learning assessment scores.

Supplementary Table S2 Self-reported cognitive load ratings across learning periods.

Supplementary Table S3 Participant-level fNIRS signal statistics.

Supplementary Table S4 Complete machine learning classification results.
